# LED-light-activated photocatalytic performance of metal-free carbon-modified hexagonal boron nitride towards degradation of methylene blue and phenol

**DOI:** 10.3762/bjnano.13.114

**Published:** 2022-11-22

**Authors:** Nirmalendu S Mishra, Pichiah Saravanan

**Affiliations:** 1 Environmental Nanotechnology Laboratory, Department of Environmental Science and Engineering, Indian Institute of Technology (ISM), Dhanbad-826004, Jharkhand, India

**Keywords:** carbon modification, hexagonal boron nitride (HBN), LED light, phenol, photocatalysis

## Abstract

The present study outlines the transformation of non-photoresponsive hexagonal boron nitride (HBN) into a visible-light-responsive material. The carbon modification was achieved through a solid-state reaction procedure inside a tube furnace under nitrogen atmosphere. In comparison to HBN (bandgap of 5.2 eV), the carbon-modified boron nitride could efficiently absorb LED light irradiation with a light harvesting efficiency of ≈90% and a direct bandgap of 2 eV. The introduction of carbon into the HBN lattice led to a significant change in the electronic environment through the formation of C–B and C–N bonds which resulted in improved visible light activity, lower charge transfer resistance, and improved charge carrier density (2.97 × 10^19^ cm^−3^). This subsequently enhanced the photocurrent density (three times) and decreased the photovoltage decay time (two times) in comparison to those of HBN. The electronic band structure (obtained through Mott–Schottky plots) and charge trapping analysis confirmed the dominance of e^−^, O_2_^−•^, and ^•^OH as dominant reactive oxygen species. The carbon modification could effectively remove 93.83% of methylene blue (MB, 20 ppm solution) and 48.56% of phenol (10 ppm solution) from the aqueous phase in comparison to HBN which shows zero activity in the visible region.

## Introduction

Hexagonal boron nitride (HBN) commonly known as white graphene belongs to a class of two-dimensional layered crystalline materials. It comprises boron (B) and nitrogen (N) atoms in equal stoichiometry in a honeycomb-like arrangement comparable to that of graphene [1–3]. Its interlayer stacking consists of a sandwich-type arrangement of B and N atoms. The N atom is more electronegative than the B atom, leading to polarization and localization of electrons in the HBN lattice. This results in photoinactivity due to a wider bandgap (5.5 eV) and limits its applicability to adsorption, drug delivery, insulators, flame retardants, hydrogen storage, among others [3–9]. This dictates the development of various innovative approaches to make it as a light-driven nanomaterial owing to its potential capabilities.

The light-driven ability of HBN can be achieved through multiple strategies. These include varying the structural morphology, heterojunction formation with a suitable photocatalyst, and doping with heteroatoms. The variation in the structural morphology results in development of multiple active sites that ensure adsorption and effective charge transfer [10]. On the other hand, HBN has been utilised as a support material in the formation of heterojunctions owing to its large surface area and separation of charge carriers due to appropriate positioning of edge potentials with respect to the synergised photocatalyst [11–13]. The process of doping includes introduction of heteroatoms such as sulphur, oxygen, and carbon into the HBN lattice. The doping of oxygen into the HBN lattice results in the generation of nitrogen vacancies and formation of energy sub-bands which help in overall reduction of the bandgap energy (*E*_g_) [14]. At the same time, the incorporation of carbon into the HBN lattice results in the delocalization of electrons with simultaneous reduction in bandgap and is directly dependent upon the percentage of carbon introduced. This demonstrates the potential of HBN to be used as a photocatalytic material. However the studies in the sense of exploring its photocatalytic ablity intented for environmental applications is very limited [15–17]. This has motivated us to extend our study on the specified subject.

The present study discusses LED light-responsive modified boron nitride (MBN) towards its photocatalytic application. The HBN was modified by introducing carbon through the solid-state reaction method. Such introduction of carbon into the HBN lattice transformed it into a good light-responsive material with improved charge carrier density (2.97 × 10^19^ cm^−3^). The LED light harvesting properties were analysed through various established characterization techniques and the photocatalysis was verified by eliminating the aqueous phase methylene blue (MB: 93.83%) and phenol (48.56%) moieties. The mechanistic insights on the transfer and separation of charge carriers along with the photodegradation performance and reactive oxygen species (ROS) trapping have been enunciated in detail. The apparent quantum efficiency (AQE) further substantiated the potential of MBN to be used as a visible light photocatalyst.

## Materials and Methods

### Chemicals required

Boric acid (H_3_BO_3_), melamine, glucose, hexagonal boron nitride nanopowder (BET surface area: 19 m^−2^ g^−1^), MB, and phenol were purchased from Alfa Aesar and TCI chemicals. All the purchased chemicals were high purity analytical grade reagents and utilized without any further purification.

### Synthesis procedure

The modified HBN was synthesized through a solid-state reaction approach with various modifications to the process described in Wang et al. [17]. An equimolar mix (0.1 M) of melamine, boric acid, and glucose was finely grounded in an agate mortar pestle to form a uniform white mixture. The bandgap could be regulated by the amount of carbon substituted into the lattice sites, with higher concentration of C atoms leading to better light harvesting and electronic properties [17–18]. Thus, the amount of glucose utilized for this study was fixed at 80 wt %. The obtained mixture was placed in a boat-type alumina crucible and subjected to heat treatment in a tube furnace at 900 °C for 3 h with a heating ramp rate of 5 °C/min under nitrogen atmosphere. Finally, the obtained MBN was naturally cooled to room temperature, washed multiple times with DI water, and dried overnight at 60 °C. The commercially available HBN was used as a control sample.

### Characterization techniques

A Rigaku Smart Lab high-resolution X-ray diffractometer (HR-XRD) equipped with a HyPix-3000 detector and Cu anode emitting Kα radiation was employed to obtain the crystallographic characterization. The morphology of the obtained nanostructures was captured by high-resolution transmission electron microscopy (HRTEM, Talos F200X G2, Thermo Scientific). The optical properties were characterized with a Shimadzu UV 2600 UV–vis spectrophotometer with an integrating sphere attachment using BaSO_4_ as the standard. The electronic arrangement of the studied materials was revealed through a PHI 5000 versa probe III high-resolution X-ray photoelectron spectroscope (HR-XPS). The mineralization efficiency was estimated through the variation in the total organic carbon content by using a Shimadzu TOC-L CSH analyser. The surface area and pore characteristics were characterized by a Micromeritics (3FLEX 3500) gas sorption analyser. The surface charge was analysed through a Zeta-Meter 4.0 (Zeta-Meter, Inc, USA). The electron paramagnetic resonance (EPR) experiments were performed by utilizing an EMX micro A200-9.5/12/S/W, Bruker Biospin, Germany.

### Photoelectrochemical study

The photoelectrochemical properties of the studied materials were evaluated through a CHI 650 electrochemical workstation comprising a three-electrode system with Pt and Ag/AgCl as counter and reference electrodes, respectively. The setup consists of a 0.5 M Na_2_SO_4_ electrolyte/hole-scavenger solution along with LED lamps as a visible light source. The working electrode was fabricated through an ITO-PTFE electrode (dimensions: 1 cm × 1 cm) drop casted with a slurry of the studied materials, isopropanol and Nafion solution. The Mott–Schottky (MS) analysis was performed at a frequency of 1 kHz while the electrochemical impedance spectroscopy (EIS) studies were conducted at 0 V DC under a frequency range of 10^5^ to 100 Hz. The open circuit potential as a function of time (OCPT) was performed under alternating light and dark conditions. The linear sweep voltammetry (LSV) studies were conducted under both dark and light conditions with a scanning speed of 5 mV/s.

### Photocatalytic activity

The LED-light-driven photocatalysis experiments were performed in a 250 mL conical flask containing 50 mg of the as-synthesized material and 200 mL of a solution containing 20 ppm of MB and 10 ppm of phenol under continuous stirring. All the batches were subjected to a dark reaction until adsorption–desorption equilibrium was achieved. A control experiment in the absence of photocatalysts was performed to study the removal percentage due to photolysis of H_2_O_2_. Aliquots were drawn at regular intervals, ultracentrifuged at 7500 rpm, and then subjected to quantification of residual concentration of pollutants using a spectrophotometer (UV 2600 SHIMADZU, Japan). The intermediates formed during the photodegradation of phenol were analysed through a gas chromatograph coupled with a mass spectrometer (GC–MS, Thermo Fisher Scientific) by taking aliquots at 150 min and 330 min. The photocatalytic degradation and mineralization efficiencies were calculated by utilizing the following equations [19]:


[1] $$E=\left\{\frac{\left[1-\text{Abs}\left(t\right)\right]}{\text{Abs}\left(0\right)}\right\}*100\%,$$

[2] $$\text{Mineralization}=\left\{\frac{1-\text{TOC}\left(t\right)}{\text{TOC}\left(0\right)}\right\}*100\%,$$

where Abs(0) symbolizes the initial absorbance, Abs(*t*) represents the absorbance of the samples at varied time intervals, TOC(0) represents the initial TOC, and TOC(*t*) denotes TOC at varied time intervals.

### Apparent quantum efficiency

The photon harvesting ability of the as-synthesized MBN was evaluated by the apparent quantum efficiency (AQE) parameter. The AQE was evaluated by using Equation 3 [20]:


[3] $$\text{AQE}=\frac{\text{Rate of reaction of the species }\left(K\left(s\right)\right)}{\text{Rate of photon absorption }\left(I\left(s\right)\right)}.$$

The intensity of the incident radiant energy was determined through a radiometer and found to be 0.05 W/cm^2^.

## Results and Discussion

### Crystallographic information and morphological analysis

**Figure 1:**
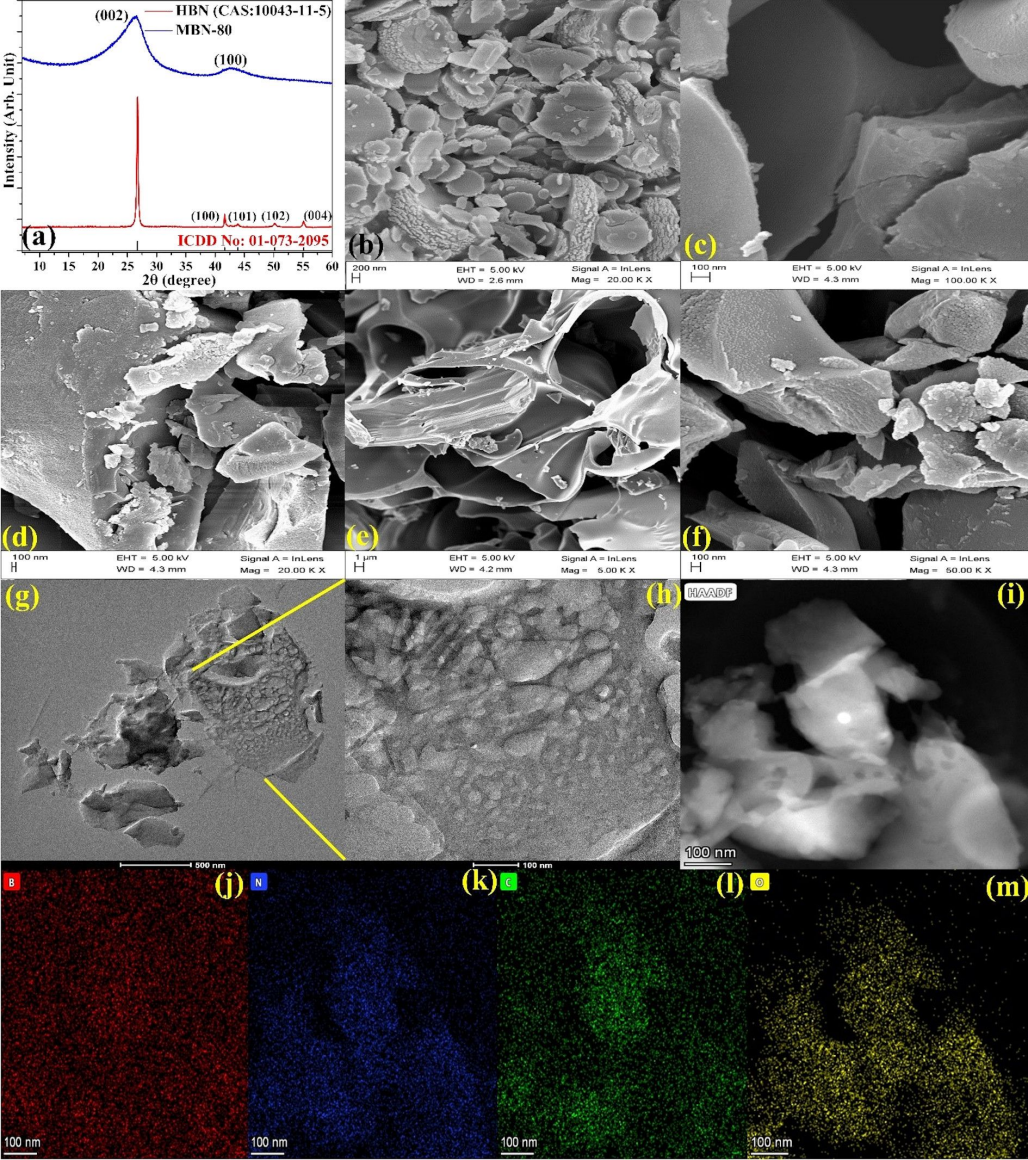
(a) HR-XRD plots for HBN and MBN-80, (b–d) SEM images for HBN, MBN-25, MBN-50, and (e, f) MBN-80. HRTEM images for (g, h) MBN-80 nanosheets, (i) HAADF STEM image, and (j–m) elemental mapping of B, N, C, and O in MBN-80.

The crystallographic information obtained for MBN is shown in Figure 1a. The HR-XRD pattern demonstrates characteristic diffraction peaks at 26.14° and 42° corresponding to (002) and (100) planes of HBN, respectively. The interlayer distance (002) for MBN was found to be 0.346 nm which is considerably greater than that of HBN (0.334 nm). This indicates the turbostratic and poor crystalline nature of the as-synthesized MBN. The characteristic peak shift and widening can be attributed to the surface modification through simultaneous replacement of nitrogen atoms with larger carbon atoms [18,21–22]. The surface morphology of HBN, MBN-25, MBN-50, and MBN-80 samples have been depicted in Figure 1b–f. Furthermore, the atomic microstructure of MBN-80 demonstrates a sheet-like porous structure with a homogeneous distribution of mesopores and can be visualized through the HRTEM images in Figure 1g–m. The formation of mesopores could be attributed to the bubbling of various gases (NH_3_, CO_2_, H_2_O) generated during the synthesis process [3,23].

### Elemental characteristics and surface area

**Figure 2:**
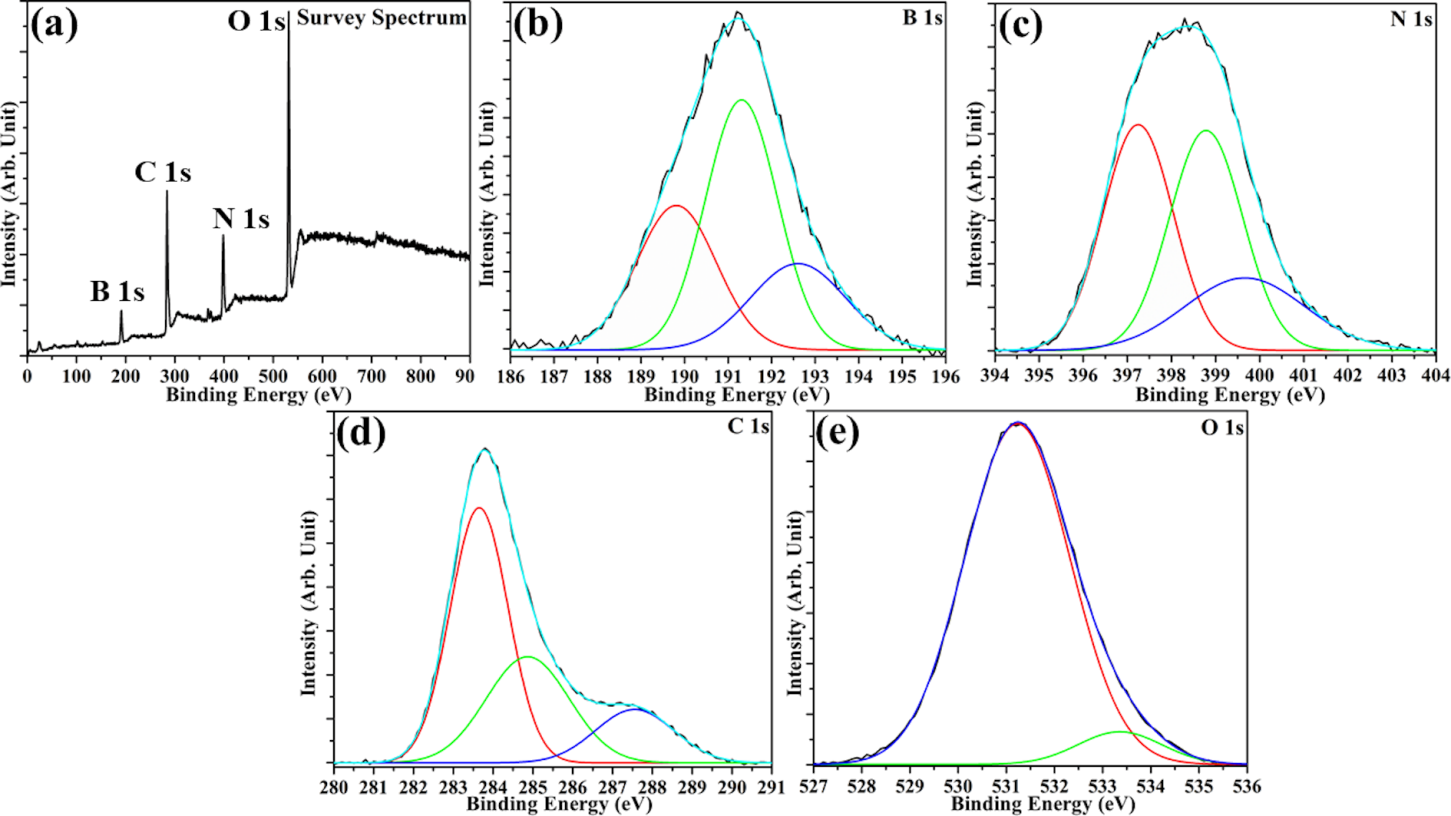
XPS binding energy spectrum for constituent elements from MBN. (a) Survey scan, (b) B 1s, (c) N 1s, (d) C 1s, and (e) O 1s.

**Figure 3:**
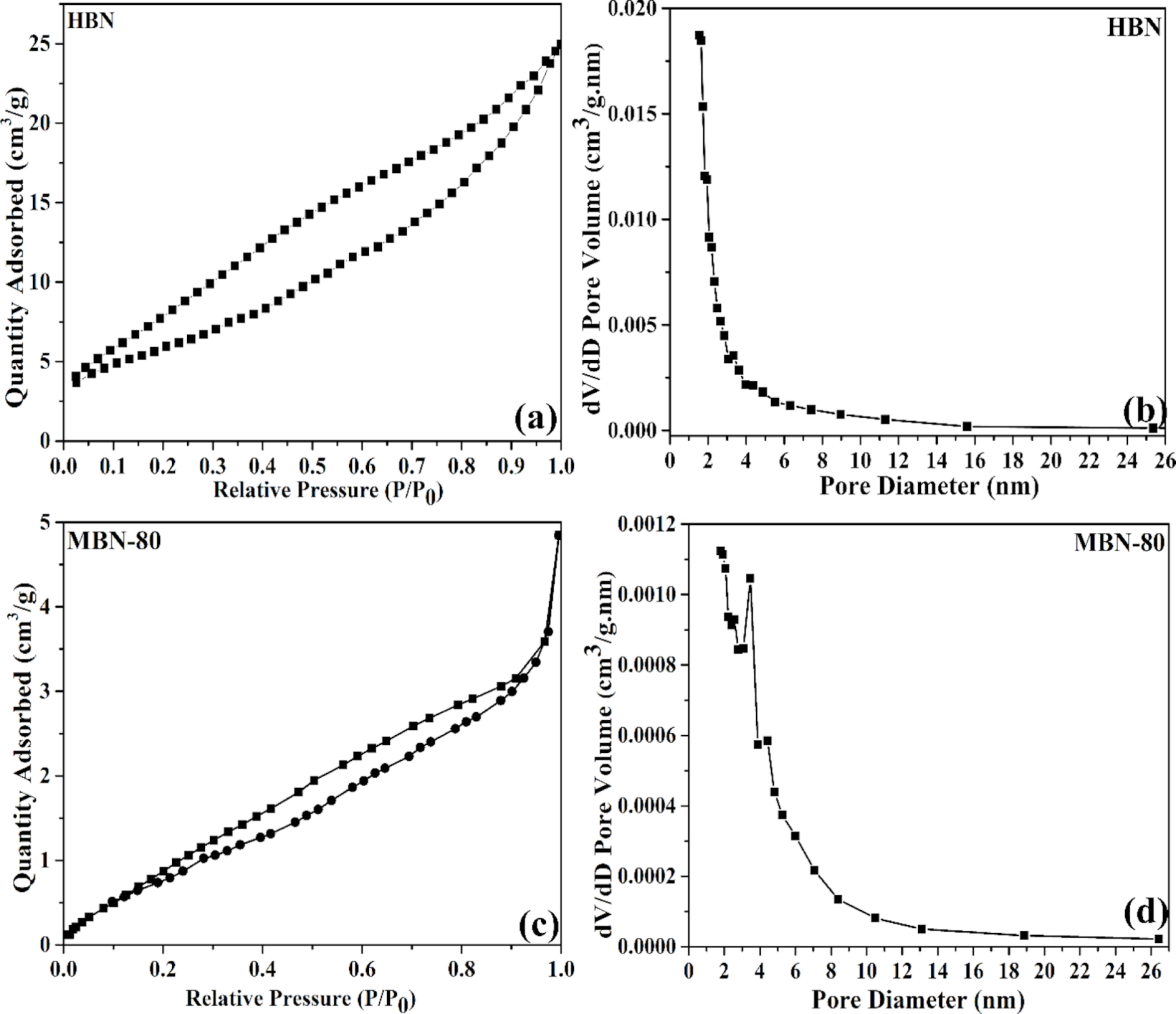
BET adsorption–desorption isotherm and BJH pore distribution for (a) HBN and (b) MBN-80.

The XPS binding energy (BE) survey spectrum for MBN and its constituent elements have been depicted in Figure 2a. The existence of B 1s, N 1s, and C 1s in the BE spectra clearly indicates the formation of the BN framework along with the introduction of carbon. The B 1s spectra seen in Figure 2b could be further deconvoluted into three subpeaks at 191.30 (B–N), 189.81 (B–C), and 192.60 eV (B–O) in MBN. The relative peak intensity of the B–N bond in the B 1s spectra was found to be higher than that of B–C/B–O bonds, indicating that MBN retained the principal BN framework. Further, the N 1s spectrum (Figure 2c) could be deconvoluted into three subpeaks at 397.3, 398.78, and 399.66 eV corresponding to N–B, N–C, and N–H bonds, respectively. The C 1s peak from MBN was deconvoluted into four subpeaks at 283.66 (C–B), 284.96 (C–C), 287.4 (C–N), and 288.55 eV (C=O) respectively and has been depicted in Figure 3d. The XPS analysis also validates the equal stoichiometry of B/N in the as-synthesized MBN. Furthermore, the C content in MBN was found to be 37.4% which is noticeably higher. The O 1s Be spectrum has been demonstrated in Figure 2e. Thus, the previous observations strongly indicate the successful introduction of carbon into the BN network [17,24].

**Figure 4:**
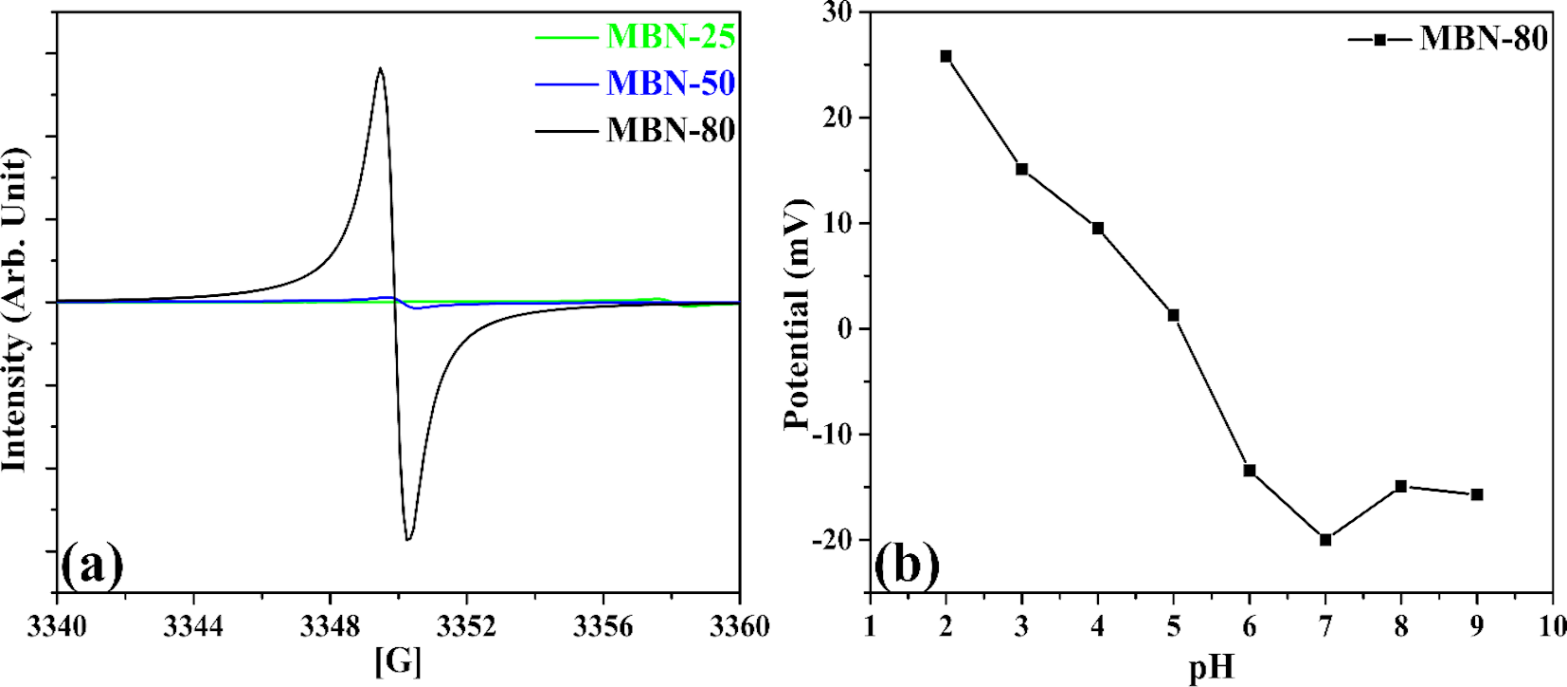
(a) EPR plot for the studied materials, (b) zeta potential study.

Additionally, the as-synthesized MBN-80 nanopowder had a lower BET surface area of 5.12 m^2^ g^−1^ with a pore volume of 0.0073 cm^3^ g^−1^. The BJH adsorption pore size distribution indicated the dominance of mesopores (2 nm < *d* < 50 nm) with an average pore size of 4.42 nm. The N_2_ adsorption–desorption isotherm demonstrated a H4 type hysteresis curve indicating narrow slit-like pores in the as-synthesized sample. In comparison, the HBN nanopowder had a BET surface area of 19.14 m^2^ g^−1^ with a pore volume of 0.0385 cm^3^ g^−1^. The BJH adsorption pore size distribution indicated an average pore size of 3.63 nm with dominance of mesopores (1.54 nm < *d* < 60 nm). The BJH adsorption pore size distribution along with the BET adsorption–desorption hysteresis for HBN and MBN-80 have been schematized in Figure 4. The BET surface area and pore volume decreased in MBN-80 and could be attributed to the structural pore collapse or excess carbon aggregation [25]. However, the increase in carbon content contributed to the improvised light utilization property.

### Electronic properties and zeta potential

The electronic properties exhibited by as-synthesized samples were also studied through light-induced EPR measurements. The Figure 4a demonstrates EPR spectra of MBN-25, MBN-50, and MBN-80. The samples exhibit single Lorentzian lines which can be attributed to the unpaired electrons. The MBN-80 sample shows the highest EPR spin intensity, meaning greater concentration of unpaired electrons along with higher electron delocalization which are highly favourable towards enhanced separation and generation of charge carriers [26–27]. Additionally, surface charge is an important parameter which governs the adsorption of the pollutant moiety over the photocatalyst. Thus, it becomes very important to study the variation of surface charge of the MBN-80. The variation of surface charge over MBN-80 in terms of pH value and isoelectric point of the solution was studied through zeta potential analysis (depicted in Figure 4b). It was observed that the surface of MBN-80 is positively charged at lower pH values which gradually increases with the increase in pH range. The isoelectric point was determined to be at pH 5.08. The MBN-80 was found to be negatively charged with a zeta potential of −20 mV at a neutral pH. This implies that the surface of MBN-80 is negatively charged above pH 5.08 which results in better adsorptive ability towards positively charged pollutant moieties and vice-versa.

### Optical studies

**Figure 5:**
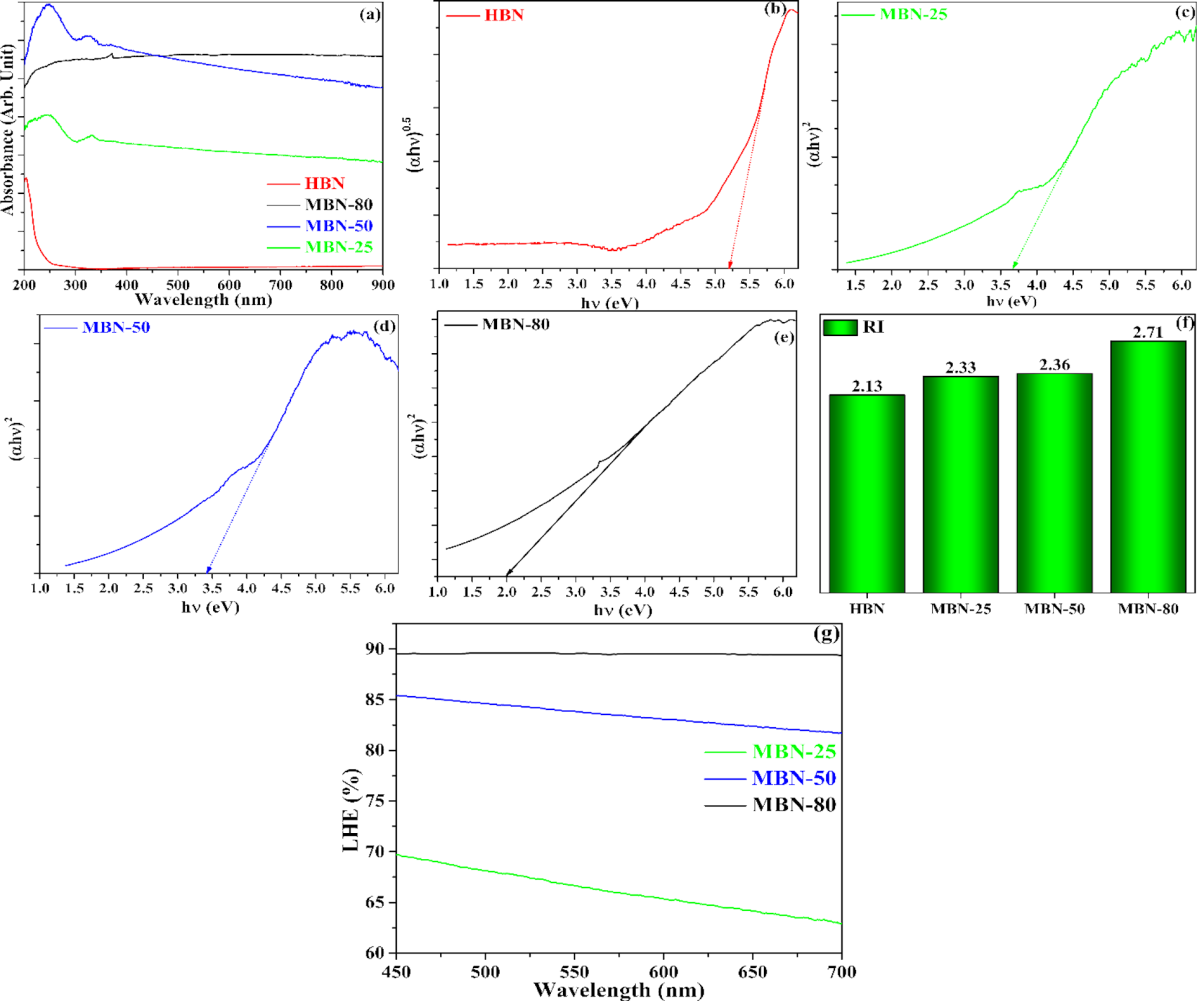
(a–e) UV-DRS and Tauc plot, (f) RI plot, and (g) LHE plot for the studied materials.

The UV–vis light harvesting characteristics (absorbance, bandgap (*E*_g_), RI, and light harvesting efficiency (LHE)) of HBN and as-synthesized MBN materials were obtained in the spectral wavelength of 200 to 900 nm and are illustrated in Figure 5. The *E*_g_ for HBN, MBN-25, MBN-50, and MBN-80 were extracted from the UV-DRS absorbance spectra through the Kubelka–Munk function by utilizing the Tauc plot mentioned in Equation 3. The obtained values were depicted in Figure 5a–e. The enhanced light absorption properties could be attributed to the grey/black colour of MBN and enhanced charge transfer attained due to the change in the electronic structure through the formation of C–N moieties in the BN framework [16].


[4] $${\left(\alpha E\right)}^{\frac{n}{2}}=A\left(E-E_{g}\right),$$

[5] $$E=\frac{hc}{\lambda },$$

where *h*, α, *c*, *E* denote the Planck’s constant, absorption coefficient derived from the Lambert’s equation, speed of light (3 × 10^8^ m s^−1^), and energy, respectively. The value of *n* (*n* = 4 for direct bandgap and *n* = 1 for indirect bandgap) depends upon the nature of the electronic transition within the semiconductor. The HBN exhibited an absorption edge at about 220 nm corresponding to a bandgap (*E*_g_) of 5.2 eV, whereas the bandgap of MBN materials was found to be 3.68, 3.41, and 2 eV for MBN-25, MNB-50, and MBN-80, respectively.

The material capability to interact with incident photons and light utilization were analysed by calculating the refractive index of the studied materials through the Moss relation (Equation 6) and was depicted in Figure 5f. In Equation 6, *E*_g_ denotes the calculated band energy from UV-DRS analysis and *R* is the refractive index [28].


[6] $$E_{\text{g}}R^{4}=108\text{ eV.}$$

Additionally, the photon harvesting ability of the studied photocatalysts was also evaluated by determining the light harvesting efficiency (LHE). The LHE of the material was determined from the following equation and has been demonstrated in Figure 5g [29].


[7] LHE%=100−T−R,

where *T* and *R* denote the transmittance and reflectance for the specified materials, respectively. It was observed that the MBN had an enhanced LHE (90%) in comparison to that of HBN with zero activity in the visible range, 85% (MBN-50), and 70% (MBN-25).

### Electrochemical analysis

**Figure 6:**
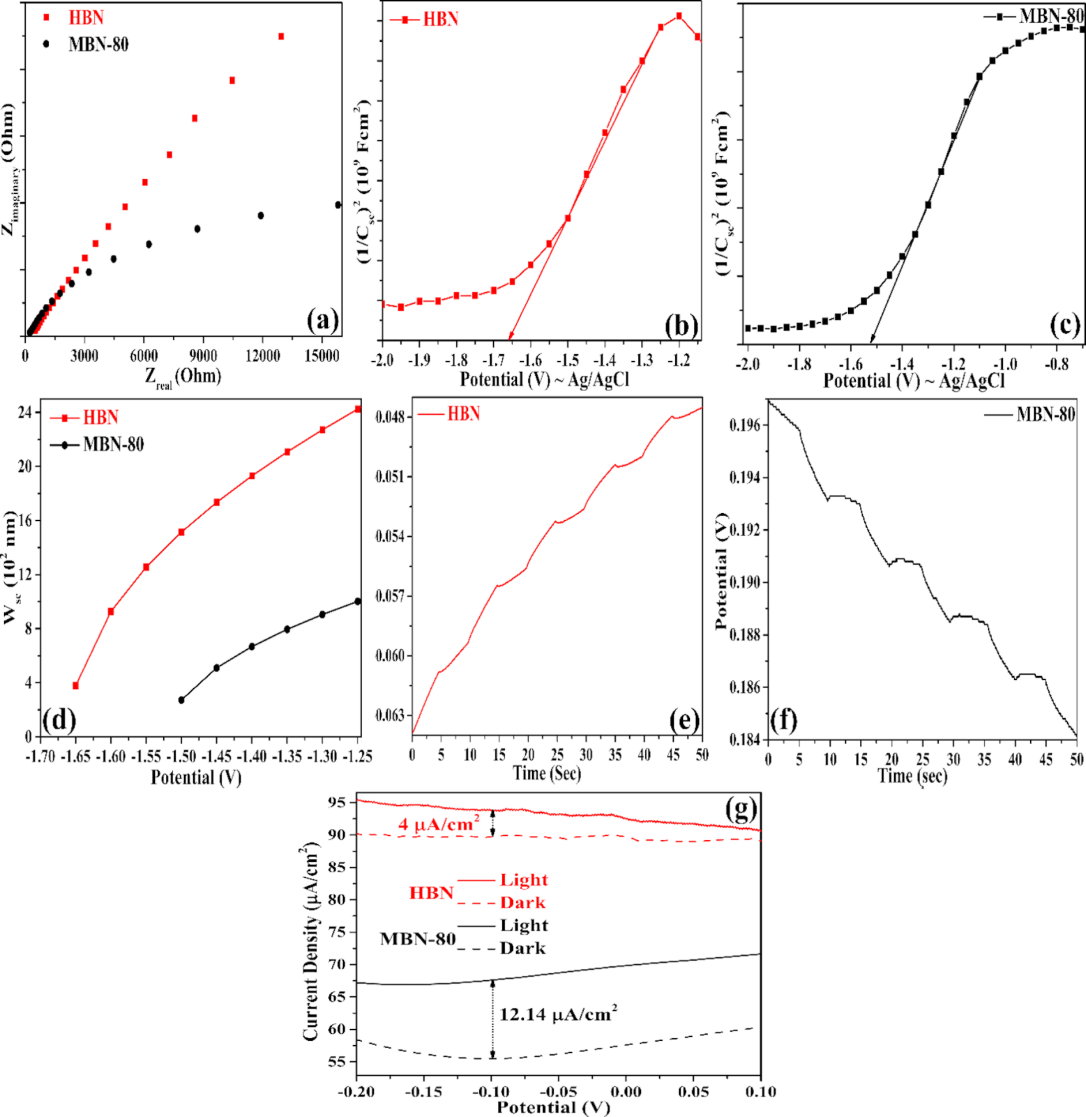
(a) Nyquist plot for HBN and MBN-80, (b, c) MS-plot, (d) *W*_sc_ analysis for HBN and MBN-80, (e, f) OCPT, and (g) LSV plots for HBN and MBN-80.

The EIS analysis provides further evidence on the enhanced performance of MBN-80 by providing in-depth information on the charge transfer kinetics, and the obtained Nyquist plots are depicted in Figure 6a. The charge transfer resistance at the electrode–electrolyte interface can be interpreted through the arc radius from the Nyquist plot [30].

The lower Nyquist radius for MBN-80 thus demonstrates the enhanced electrochemical performance and lower charge transfer resistance. This mainly means an enhanced electron transfer from MBN-80 for a favourable visible light photocatalysis.

Additionally, the capacitance of the electrical double layer generated at the semiconductor–electrolyte interface was measured as a function of the applied potential (*E*_appl_) and enunciated through Equation 8. Furthermore, the band structure, Debye length (*L*_DB_), density of charge carriers (*N*_d_), and width of the space charge region (*W*_sc_) pertaining to MBN-80 could also be calculated from the Mott–Schottky analysis through the following equations [28,31–32].


[8] $$\frac{1}{C_{\text{sc}}^{2}}=\frac{2}{eA^{2}N_{d}\varepsilon \varepsilon_{0}}\left(E_{\text{appl}}-E_{\text{FB}}-\frac{k_{\text{B}}T}{e}\right)$$

[9] $$N_{\text{d}}=\frac{2}{e\varepsilon \varepsilon_{0}A^{2}}\frac{1}{\frac{d}{dE_{\text{appl}}}\left(\frac{1}{C_{\text{sc}}^{2}}\right)}$$

[10] $$L_{\text{Debye}}=\sqrt{\frac{\varepsilon \varepsilon_{0}k_{\text{B}}T}{e^{2}N_{\text{d}}}}$$

[11] $$W_{\text{sc}}=\sqrt{\frac{2\varepsilon \varepsilon_{0}\left(E_{\text{appl}}-E_{\text{FB}}\right)}{eN_{\text{d}}}}$$

where *C*_sc_, *e*, *A*, ε, ε_0_, *k*_B_, and *T* indicate the capacitance of the space charge region, charge of an electron, active area of the electrode, dielectric constant, permittivity of free space, Boltzmann’s constant, and absolute temperature, respectively.

The MS plots for the studied materials is depicted in Figure 6b,c. The n-type nature of the materials was confirmed through the occurrence of a positive slope. Subsequently, the flat band potential (*E*_FB_) was determined by extrapolating the linear portion of the MS plot [33]. The *E*_FB_ values for HBN and MBN-80 were determined to be −1.66 V and −1.52 V, respectively. The respective potentials vs Ag/AgCl were converted to NHE.

The CB_MBN_ shifted to a lower edge potential (by 0.14 V) due to the interaction with the adjacent nitrogen/boron atoms through C–B and C–N bonds [17]. Furthermore, this also increased the availability of charge carriers in MBN-80 (*N*_d_: 2.97 × 10^19^ cm^−3^) which was found to be significantly greater than that in HBN (*N*_d_: 7.38 × 10^18^ cm^−3^). The increased charge density in MBN could be attributed to the accumulation of electrons over the carbon atom which aids in the photocatalytic process [24]. The MBN-80 also exhibited a lower *L*_DB_ and charge transit time through the depletion layer (charge transit time ∝ (*L*_Debye_)^2^), meaning a lower recombination of the charge carriers leading to an enhanced photocatalytic activity [29]. The same could again be justified through the width reduction of the space charge region as seen in Figure 6d. Such reduction results in rapid transport and separation of the charge carriers in comparison to HBN.

The electrochemical and potentiodynamic characteristics of MBN-80 were studied by analysing LSV and OCPT data. The LSV response for MBN-80 was studied under both visible and dark conditions, whereas the OCPT studies were performed under a constant voltage and discrete light illumination cycles [34]. The LSV and OCPT plots were depicted in Figure 6e–g. It was observed that the illumination of MBN-80 generated electron–hole pairs which resulted in a voltage buildup which subsequently decayed upon quenching of the light source [31]. The obtained photovoltage plots were subsequently fitted with an exponential decay curve. The MBN-80 demonstrated a reduced decay time (4.55 s) in comparison to that of HBN (2.65 s) emphasizing an efficient charge separation. The photovoltage was measured to be 0.198 V for MBN-80 which is about 3.3 times more than that of HBN (0.06 V), depicting the superiority of MBN-80. Further, it generated a photo current density of 12.14 µA/cm^2^ which is three times greater than that of HBN (4 µA/cm^2^).

### Photocatalytic study of modified boron nitride

#### Photocatalytic study

**Figure 7:**
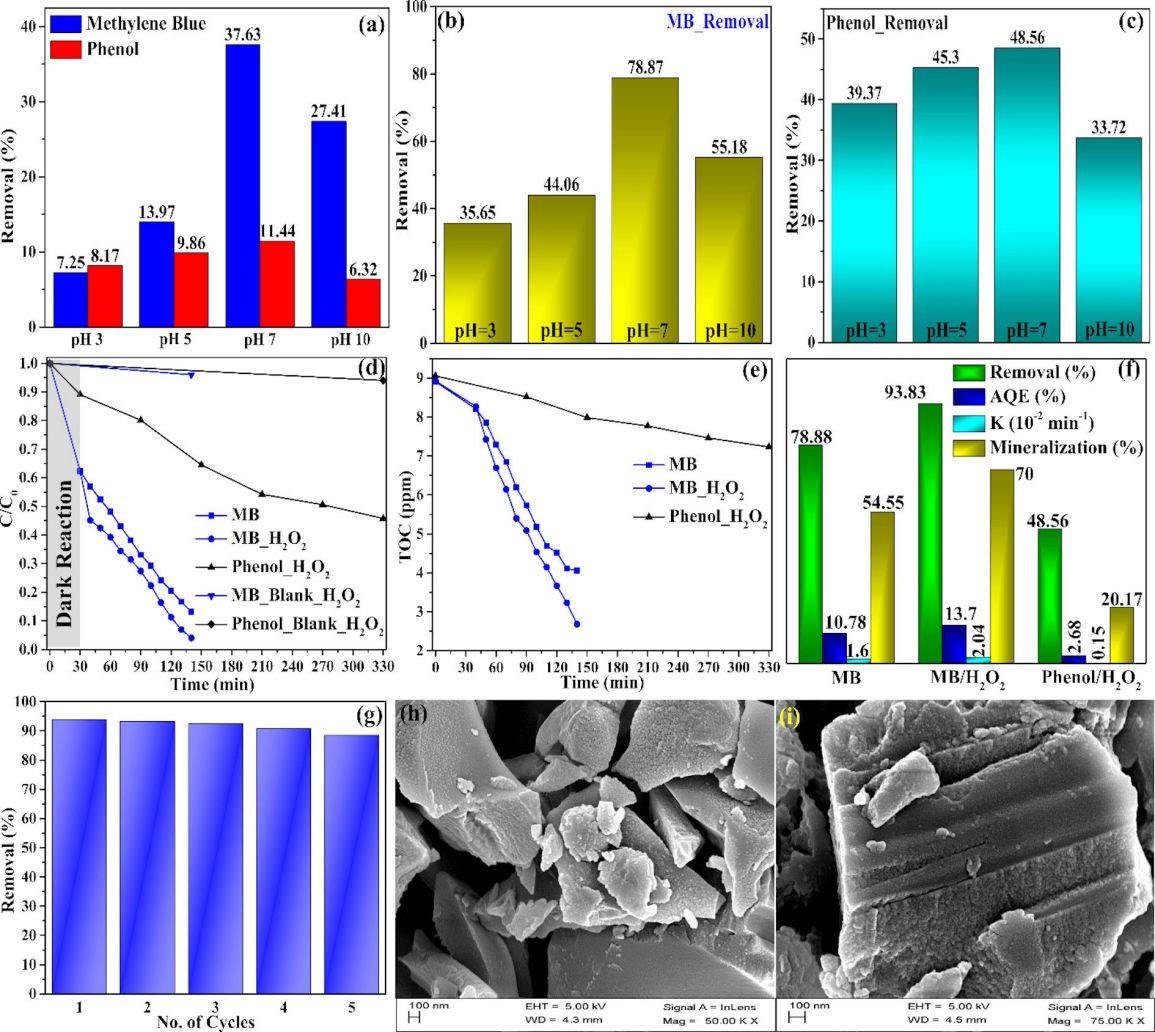
(a) Adsorption of MB and phenol at varied pH values. (b) The effect of pH variation on photocatalytic degradation of MB. (c) The effect of pH variation on photocatalytic degradation of phenol. (d) LED photocatalysis over MBN-80 on MB (20 ppm) and phenol (10 ppm). (e) Mineralization of MB and phenol, (f) degradation (%), AQE, rate constant (*K*) and mineralization efficiency (%), and (g) photocatalytic performance of MB up to five cycles. (h, i) SEM images of MBN-80 before and after five reuse cycles.

Figure 7a–g depicts the adsorption and photocatalytic degradation performance of MBN-80 towards MB (20 ppm) and phenol (10 ppm) accomplished through LED irradiation. Specifically, it removed 78.87% of MB (20 ppm solution) which was further enhanced to 93.83% in the presence of 0.05 M H_2_O_2_. The photocatalysis process followed a pseudo-first-order reaction kinetics with a rate constant (*K*) of 0.016 min^−1^ (for the aqueous phase MB) and 0.0204 min^−1^ (for the aqueous phase MB in the presence of H_2_O_2_) with a TOC removal of 54.55% and 70%, respectively. On the other hand, the photocatalytic degradation of phenol demonstrated a removal rate of 0.0015 min^−1^ with 48.56% removal and a mineralization efficiency of 20.17%. The AQE for phenol/H_2_O_2_ system was found to be at 2.67%. A negligible removal percentage in the order of 4% for MB and 6% for phenol over a period of 140 min and 330 min, respectively, was observed for control experiments.

The photocatalytic degradation of MB was found to be dependent on the solution pH value with greater removal at higher pH values. The variation in the photocatalytic degradation as a function of pH was depicted in Figure 7b. The high removal rate towards MB could be ascribed to a better adsorption boosted through electrostatic interactions (cationic MB and negatively charged surface of MBN-80) and the synergy obtained through the photoactivity of MBN-80 and dye sensitization through MB moieties. The photosensitization of MB dye moieties generates additional photo-excited electrons which are transferred into the CB of MBN-80, resulting in enhanced charge carrier density and generation of ^•^OH via the O_2_ reduction pathway, thus enhancing dye degradation.

Conversely, the photocatalytic degradation of phenol showed a 48.56% removal with a mineralization efficiency of 20.17% and AQE of 2.67%. This could be explained by the fact that the interaction of the photocatalyst with a specified pollutant varies with respect to its chemical behaviour. In the case of MB, an electrostatic interaction was observed (owing to the negatively charged MBN-80 and positively charged MB dye moieties) and that triggered the photocatalysis reaction.

In the case of phenol, it contains a phenyl group (–C_6_H_5_) with a neutral charge and thus the interaction between this group and the photocatalyst is limited. Also, the phenol is not a photo-sensitizing compound as MB. The variation in the photocatalytic degradation as a function of pH was depicted in Figure 7c. It can be clearly observed that the degradation efficiency decreases at higher pH values (pH 10) due to changes in surface charges of the phenol moieties (*P*_ka_ = 9.3) [35]. Nevertheless, the obtained MBN-80 was found to be a potential photocatalyst which can be activated with LED light and has a limited spectrum as compared to solar light. The SEM images of MBN-80 before and after five reuse cycles is depicted in Figure 7e,f. A comparison between the photocatalytic performance of MBN-80 and other photocatalytic materials reported in the literature is discussed in Table 1.

**Table 1:** A comparison of the photocatalytic performance of MBN-80 with previously published photocatalytic materials.

Sl.No	Photocatalyst	Light source	Pollutant/degradation efficiency	Ref.

1	BCN-80	300 W xenon lamp with 420 nm cutoff filter	U (VI)/97.40%	[17]
2	Ag_2_CrO_4_ / BN composite photocatalyst	visible light	rhodamine B/96.70%	[12]
3	Bi_4_O_5_I_2_/3 wt % BN composite photocatalyst	300 W xenon lamp	rhodamine B/80% bisphenol-A/97%	[36]
4	0.9 % g-BN/g-C_3_N_4_ composite photocatalyst	300 W xenon lamp with 420 nm cutoff filter	BPA/91.9%	[37]
5	HBN-S	300 W xenon lamp	2,4-dichlorophenol/77%	[38]
6	rGO/Fe_3_O_4_/ZnO composite photocatalyst	tungsten halogen	methylene violet/83%	[39]
7	Fe_3_O_4_/ZnO/pumice composite photocatalyst	green LED	rhodamine B/72%	[40]
8	Fe_3_O_4_/ZnO/CoWO_4_ composite photocatalyst	LED	MB/99%	[41]
9	HBN/titania composite photocatalyst	UV light with a wavelength of 365 nm	rhodamine B/98% MB/92%	[42]
10	5 wt % hBN/Fe_e_O_3_ composite photocatalyst	250 W tungsten-halogen lamp	MB/91%	[11]
11	CaTiO_3_/BNQDs composite photocatalyst	sunlight	tetracycline/88.5%	[43]
12	MBN-80	LED light	MB/93.83% phenol/48.56%	present study

#### Photocatalytic mechanism

**Figure 8:**
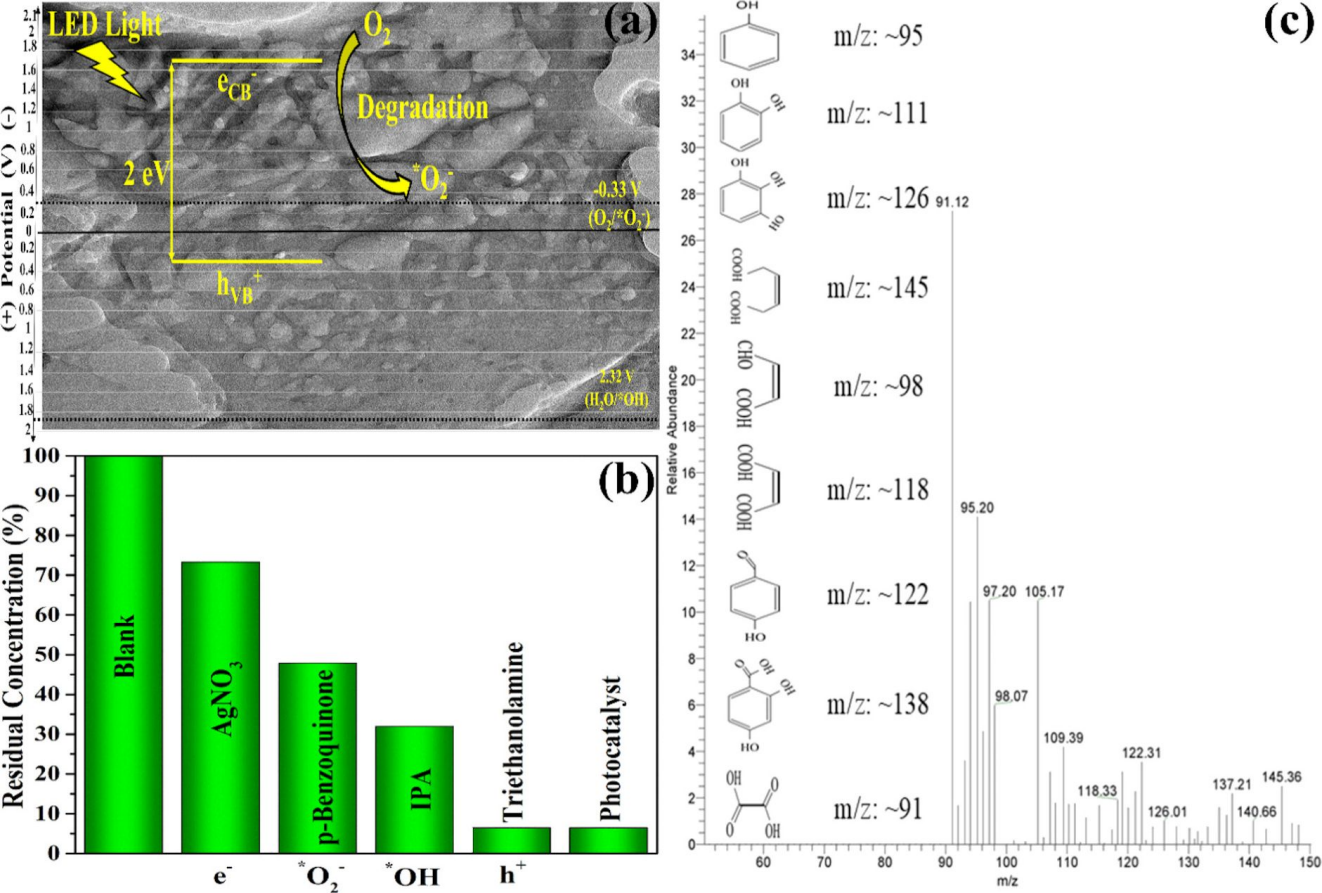
(a) Electronic band structure demonstrating the edge potentials for MBN. (b) Charge trapping analysis using various quenching reagents and (c) GC–MS analysis for the obtained intermediates.

The detailed photocatalytic mechanism is explained in Figure 8a,b. The CB potential of MBN-80 (−1.7 V vs NHE) is more negative than that of O_2_/O_2_^−•^ (−0.33 V vs NHE) [44]. Conversely, the VB potential of MBN (0.3 V vs NHE) is less positive than that of H_2_O/^•^OH (2.32 V vs NHE) [45]. This implies that the degradation could be dependent upon e^−^, h^+^ and O_2_^−•^ as the predominant ROS species. Thus, the charge trapping analysis (CTA) was conducted to determine the dominant ROS species taking part in the degradation mechanism.

The CTA was achieved by taking 1 mmol of p-benzoquinone, isopropyl alcohol (IPA), AgNO_3_, and triethanolamine (TEA) as quenching agents to trap O_2_^−•^, ^•^OH, e^−^, and h^+^, respectively [46]. The outcome of CTA was depicted in Figure 8b. The addition of TEA demonstrated an insignificant effect on the photocatalytic efficiency, which means no participation of h^+^ in the degradation process. Further, the addition of IPA also affected the degradation performance suggesting the contribution of the ^•^OH radical (from photolysis of H_2_O_2_) in the photocatalytic process. Furthermore, a substantial reduction in the photocatalytic activity was observed when AgNO_3_ and p-benzoquinone were added into the reaction system. The introduction of AgNO_3_ resulted in the capture of electrons from the reaction mixture which disrupts the generation of O_2_^−•^ through the O_2_/O_2_^−•^ reduction pathway. The same can also be observed during the addition of p-benzoquinone, which leads to a significant decrease in the photo catalytic efficiency due to scavenging of O_2_^−•^. Further, the byproducts of phenol degradation analysed through GC–MS is depicted Figure 8c. The intermediate products were identified as catechol (*m*/*z*: 111), 4-hydroxybenzaldehyde (*m*/*z*: 122), salicylic acid (*m*/*z*: ≈138), benzene-1,2,3-triol (*m*/*z*: ≈126), maleic acid (*m*/*z*: ≈118), (*Z*)-4-oxobut-2-enoic acid (*m*/*z*: ≈98), (*Z*)-hex-3-enedioic acid (*m*/*z*: ≈146), and oxalic acid (*m*/*z*: ≈91) [47–48].

## Conclusion

The comprehensive analysis of the obtained experimental outcomes substantiates MBN as a superior photoactive material with enhanced LHE, charge carrier generation, and reduced exciton recombination in comparison to HBN. The introduction of carbon into the HBN lattice sites boosted the overall photocatalytic performance through changes in the electronic structure by the formation of C–B and C–N moieties. This also led to the delocalization of electrons and accumulation of additional electrons from the graphitic carbon leading to an increase in charge carrier density within MBN-80. The removal of MB and phenol demonstrated LED-light-driven photocatalytic activity of MBN-80 over the nonresponsive photoinactive HBN.
